# The social determinants of national tuberculosis incidence rates in 116 countries: a longitudinal ecological study between 2005–2015

**DOI:** 10.1186/s12889-023-15213-w

**Published:** 2023-02-15

**Authors:** Fiona A. Költringer, Kristi Sidney Annerstedt, Delia Boccia, Daniel J. Carter, William E. Rudgard

**Affiliations:** 1 Department of Global Public Health, WHO Collaborating Centre On Tuberculosis and Social Medicine, Karolinska Institutet, Stockholm, Sweden; 2 Department of Global Health and Development, London School of Hygiene and Tropical Medicine, London, UK; 3 Department of Social Policy and Intervention, University of Oxford, Oxford, UK

**Keywords:** Tuberculosis incidence, Social determinants of health, Sustainable development goals, End tuberculosis, Ecological, Multi-country

## Abstract

**Background:**

Accelerating declines in tuberculosis (TB) incidence is paramount for achieving global goals set for 2030 by the Sustainable Development Goals and the End TB Strategy. The aim of this study was to identify key country-level social determinants of national TB incidence trends.

**Methods:**

This longitudinal ecological study used country-level data extracted from online databases from the period 2005–2015. We used multivariable Poisson regression models allowing for distinct within- and between-country effects to estimate associations between national TB incidence rates and 13 social determinants of health. The analysis was stratified by country income status.

**Results:**

The study sample included 48 low- and lower-middle-income countries (LLMICs) and 68 high- and upper-middle income countries (HUMICs), with a total of 528 and 748 observations between 2005–2015, respectively. National TB incidence rates declined in 108/116 countries between 2005–2015, with an average drop of 12.95% in LLMICs and 14.09% in HUMICs. Between LLMICs, higher Human Development Index (HDI), social protection spending, TB case detection, and TB treatment success were associated with lower TB incidence. Higher prevalence of HIV/AIDS was associated with higher TB incidence. Within LLMICs, increases in HDI over time were associated with lower TB incidence rates. Between HUMICs, higher HDI, health spending, and diabetes prevalence were associated with lower TB incidence, whereas higher prevalence of HIV/AIDS and alcohol-use were associated with higher TB incidence. Within HUMICs, increases in HIV/AIDS and diabetes prevalence over time were associated with higher TB incidence.

**Conclusions:**

In LLMICs, TB incidence rates remain highest in countries with low human development, social protection spending and TB programme performance, and high rates of HIV/AIDS. Strengthening human development is likely to accelerate declines in TB incidence. In HUMICs, TB incidence rates remain highest in countries with low human development, health spending and diabetes prevalence, and high rates of HIV/AIDS and alcohol use. Here, slowing rising rates of HIV/AIDS and diabetes is likely to accelerate declines in TB incidence.

**Supplementary Information:**

The online version contains supplementary material available at 10.1186/s12889-023-15213-w.

## Background

Tuberculosis (TB) remains the world’s biggest infectious killer, claiming an estimated 1.6 million global deaths in 2021 [1]. The World Health Organization’s (WHO) End TB Strategy aims to achieve an 80% drop in new cases of TB and 90% reduction in TB mortality by 2030 [2]. However, progress remains well short of what is necessary to achieve these goals [1, 2]. Global estimates updated to reflect disruptions to essential TB services during COVID-19 estimate that TB incidence increased by 3.6% for the first time in decades between 2020 and 2021 [1].

Action on poverty and associated risk factors is expected to play an important role in accelerating the decline in TB incidence and has been integrated as a central paradigm of the End TB Strategy [2, 3]. TB disease disproportionately affects poor and marginalised populations [4] and is strongly associated with living or working in an environment with high TB prevalence [5, 6], overcrowding [7], poor ventilation [7], malnutrition [8] and health conditions that impair host immune defence [4, 9].

**Fig. 1:**
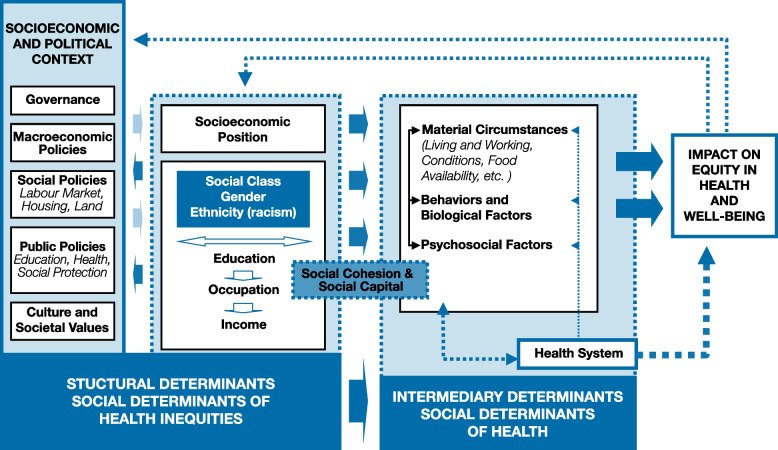
CSDH conceptual framework for action on the social determinants of health [10]

The economic and social conditions that influence TB risk are collectively known as the social determinants of health. Based on the WHO’s Commission on Social Determinants of Health (CSDH), social determinants of health can be divided into structural and intermediary determinants [10]. Factors at the structural level make-up the socioeconomic and political context as well as the individual socioeconomic position that determine people’s exposure to intermediary determinants [10]. Factors at the intermediary level include material circumstances, behaviours, biological, and psychosocial factors that have a direct impact on health outcomes, such as exposure to indoor air pollution [10]. The CSDH conceptual framework is helpful for understanding how TB occurrence may be determined by social determinants of health at the structural and intermediary levels of influence (Fig. 1).

Although improved diagnosis and treatment through national TB programmes has been linked to lower TB mortality, their impact on TB incidence remain unclear [3, 11, 12]. Growing evidence supports the need for primary disease prevention to achieve definitive reductions in TB incidence and mortality by 2035 [13–16]. In an early study in 2009, Dye et al. identified the national Human Development Index (HDI), under-five mortality rate (U5M), and access to improved sanitation services as dominant predictors of global TB incidence trends between 1997 and 2006 [17]. Since then, several studies have investigated the potential of anti-poverty strategies to accelerate progress on ending TB [18, 19]. Results consistently point to the large impact that such measures could have for TB control.

Observed changes in TB associated health risks since 2006 such as diabetes prevalence [20, 21] and undernourishment [21, 22] highlight the need for an updated analysis of the key determinants of global TB incidence trends. Extending Dye et al.’s study to consider the full period of the millennium development goals (MDGs), we aimed to investigate which social determinants of health may hold promise for accelerating declines in TB incidence. The aim of the study was to identify key social determinants of health that influenced global TB incidence trends 2005–2015. Our objectives were to 1) describe trends in TB incidence between 2005 and 2015, 2) evaluate between countries how social determinants of health are associated with TB incidence rates, and 3) evaluate within countries how trends in social determinants of health are associated with declines in TB incidence rates. Because trends in TB incidence rates differ significantly across country-income groups, we stratified our analysis into high- and upper-middle income countries (HUMICs), and low- and lower-middle income countries (LLMICs).

## Methods

### Study design and data

The study used an ecological, longitudinal design to evaluate associations between national TB incidence rates and 13 selected indicators of social determinants of health. The study period corresponded to the 11-year MDG era, 2005–2015. Data on national TB incidence rates and social determinants of health for these years were downloaded from public online data repositories in 2020. The study was reported according to STROBE reporting guidelines A.1 [23].

### Study sample

The study sample was countries with available TB incidence data in the first year of the MDG period, 2005. Countries with an annualized change in TB incidence rate greater than three standard deviations of the mean were considered outliers with unusual conditions and excluded (*N* = 1, Ethiopia) [24]. We hypothesised that different public health priorities, healthcare systems, and socioeconomic contexts could mean that the social determinants of TB incidence rates would differ by country income status. We originally grouped countries into two categories, high-income countries versus low- and middle-income countries, using World Bank income classifications from 2005 [25]. However, during the peer-review process we observed that there was a risk of modelling error from too few observations in the high-income country group. Further analysis in Additional file 1: Appendix A.2 showed that average TB incidence rates for 2005 in upper-middle income countries (70.94 per 100,000) were more similar to high-income countries (9.37 per 100,000) than lower-middle income countries (217.35 per 100,000). Grouping upper-middle income countries with high-income countries also resulted in a more equal split in observations, which fulfilled the rule of thumb of at least 10 observations per variable included in our final regression models for both groups. Therefore, our final analysis was grouped into two categories, high-income countries with upper-middle income countries, and low-income countries with lower-middle income countries. Lists of included and excluded countries are provided in the Additional file 1: Appendix A.3 and A.4.

### Study variables

The study outcome was age- and sex-standardized national TB incidence. Seventeen indicators of social determinants of TB incidence rate were identified and considered for inclusion in the study based on the CSDH framework and their availability in five public online data repositories: The World Bank Database, the Global Burden of Disease (GDB) Study, the Human Development Report (HDR), the International Labour Organization database (ILO), and the WHO TB database. All seventeen indicators were continuous variables. Two indicators were considered proxies of TB programme performance and quality (TB case detection rate, TB treatment success rate). We expected a certain degree of collinearity between indicators of socioeconomic development and assessed intercorrelations between indicators of social determinants of health using Pearson correlation. We found HDI, U5M, access to hygiene, access to drinking water, and access to clean cooking technologies to be highly correlated at ρ > 0.8 (Additional file 1: Appendix A.4, A.5) [26]. Among these indicators, HDI was selected as the most comprehensive measure tracking human development. This meant that 13/17 of the original indicators were included in the analysis. We provide a description of each variable considered for inclusion in Table 1, and fuller description of those included in the study in Additional file 1: Appendix A.6.

**Table 1:** Social determinants of health identified from five public online data repositories and considered for inclusion in the study

**Social determinant of health**	**Data source**	**Selected for analysis**^a^
Human Development Index^b^	HDR	Included
Public social protection expenditure, % of GDP	ILO	Included
Current health expenditure, % of GDP	World Bank	Included
Labour force participation rate, % of total population aged 15–64	World Bank	Included
Under-five-mortality rate, per 1000 live births^c^	World Bank	Highly correlated with Human Development Index and excluded
Population with access to clean fuels and technologies for cooking, %^c^	World Bank	Highly correlated with Human Development Index and excluded
Population using basic drinking water sources, %^c^	World Bank	Highly correlated with Human Development Index and excluded
Population using basic sanitation services, %^c^	World Bank	Highly correlated with Human Development Index and excluded
Prevalence of undernourishment, %	World Bank	Included
Prevalence of HIV/AIDS, per 1,000 (age-standardized, both sexes)^d^	GBD	Included
Prevalence of diabetes, per 1,000 (age-standardized, both sexes)^d^	GBD	Included
Prevalence of alcohol use disorder, per 1,000 (age-standardized, both sexes)^d^	GBD	Included
Prevalence of daily smoking, per 1,000 (age-standardized, both sexes)^d^	GBD	Included
Out-of-pocket expenditure, % of current health expenditure	World Bank	Included
TB case detection rate, % (all forms)	WHO	Included
TB treatment success rate, % (all new cases)	WHO	Included
Population living in urban areas, %	World Bank	Included

*Abbreviations*: *HIV/AIDS* Human Immunodeficiency Virus/Acquired Immunodeficiency Syndrome, *GDP* Gross Domestic Product, *TB* Tuberculosis, *WHO* World Health Organization, *GBD* Global Burden of Disease Study, *ILO* International Labour Organization, *HDR* Human Development Report

^a^We expected a certain degree of collinearity between indicators of socioeconomic development and assessed intercorrelations between indicators of social determinants of health using Pearson correlation

^b^For statistical analysis, HDI was multiplied by 100 to ease interpretation of results

^c^Variable was not included in statistical analysis due to high intercorrelation with HDI ρ > 0.8

^d^For statistical analysis, prevalence was converted into a rate per 1,000 population

### Missing data

We used linear interpolation and extrapolation to substitute missing observations between 2005–2015. Observed data from 2016 or 2017 was used to interpolate missing observations in 2015. Data on public social protection expenditure for Nicaragua and Sierra Leone could not be extrapolated since the observed values in 2005 and 2015 were identical. Overall characteristics of interpolated and non-interpolated data are provided in the Additional file 1: Appendix A.7.

### Data analysis

First, we summarised trends in TB incidence rates as the absolute and percentage change between 2005–2015 using mean and standard deviation (SD). We also described average values of our 13 indicators of social determinants of health in 2005, 2015, and between 2005–2015 using mean and SD. For pooled observations between 2005–2015 we also reported between- and within-country components of the overall SD. Student’s t-test was performed to assess differences in the mean values of social determinants of health between our two categories of country based on World Bank country-income classifications.

Second, we evaluated associations between TB incidence rates and our 13 social determinants of health using univariable and multivariable random effects within-between Poisson regression models. The within-between modelling approach provided the ability to distinguish differing relationships between TB incidence and social determinants based on within- and between-country variation over time [27]. We provide an example interpretation of these two types of variation using HDI. Hypothesizing that this social determinant of health would be associated with lower TB incidence rates, between-country analysis would test whether countries with higher values of HDI have lower national TB incidence rates; whereas within-country analysis would test whether within the same country, years with higher than average HDI have lower TB incidence rates. The significance level was set to 5% and results were reported as incidence rate ratio (IRR). Analyses were carried out in Stata 15.1. The analysis code and data are available online (https://osf.io/x6uag/).

### Sensitivity analysis

In a sensitivity analysis, we evaluated multivariable associations between TB incidence rates and more distal indicators of social determinants of health, which included HDI, public social protection spending, current health expenditure, and labour force participation rates. This was to rule out possible attenuation of associations between more distal social determinants of health and TB incidence by intermediary social determinants of health lying on the causal pathway.

## Results

### Study sample

**Fig. 2:**
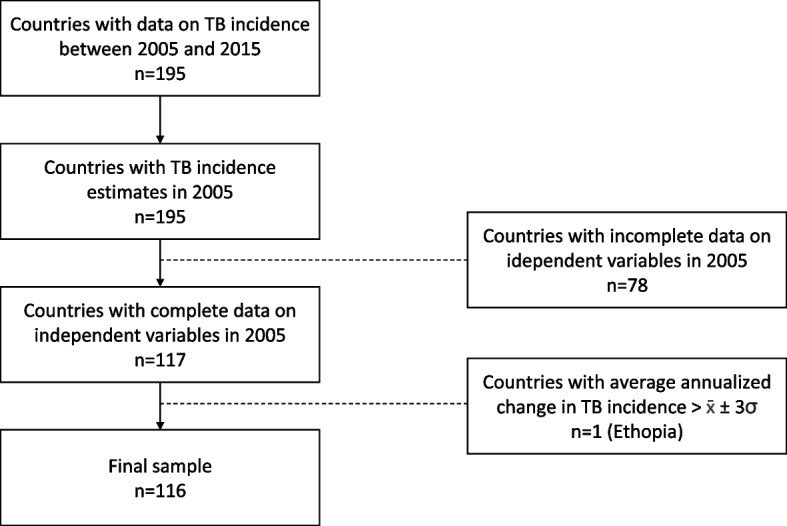
Sample size selection and inclusion criteria for countries

Of 195 countries with TB incidence rate data in the GBD study, incomplete data in 2005 excluded 53 HUMICs and 24 LLMICs. Ethiopia was excluded due to outlying annualized change in TB incidence rate (Fig. 2). Among the 116 included countries, 48 (41%) were LLMICs and 68 (59%) were HUMICs. The final sample included 24 of the 30 countries defined by the WHO as having a high TB burden and represents 68% of all estimated incident TB cases worldwide [1].

### Summary of trends in TB incidence rates between 2005–2015

Across all countries, the mean TB incidence rate in 2005 was 134.77 per 100,000 population (SD:155.44, IQR:220.65). In LLMICs, the mean TB incidence rate in 2005 was 249.57 per 100,000 population (SD:144.74, IQR:165.61), and in HUMICs it was 53.74 per 100,000 population (SD:103.28, IQR:40.51). Overall, TB incidence rates between 2005–2015 declined in 108 of the 116 included countries (93%). Among the eight countries where TB incidence rates increased between 2005–2015, two were LLMICs and six were HUMICs. In LLMICs, the mean change in TB incidence rate between 2005–2015 was -29.48 per 100,000 (SD:25.44, IQR:29.53), and in HUMICs, it was -9.00 per 100,000 population (SD:15.70, IQR:6.70). In LMICs, this corresponded to a mean percentage decline in TB incidence of 13.60%, and in HUMICs, a decline of 20.95%. Descriptive statistics for our 13 social determinants of health in 2005, 2015, and between 2005–2015 are summarized in Table 2. Results of univariable regression analysis are presented in Table 3.

**Table 2:** Descriptive statistics of social determinants of health in 2005, 2015, and between 2005–2015

	**2005** (*N* = 116; Obs = 116)		**2015** (*N* = 116; Obs = 116)		**2005–2015** (*N* = 116; Obs = 1,276)				
**Social determinants**	Mean	SD	Mean	SD	Mean	SD	SD-within	SD-between	*p*^a^
**HDI**^b^									< 0.001
HUMIC	75.43	6.42	75.54	6.64	78.90	8.32	1.70	8.20	
LLMIC	45.48	7.47	46.56	8.88	53.08	9.73	2.37	9.53	
**Public social protection expenditure, % of GDP**									< 0.001
HUMIC	10.83	6.00	10.90	6.10	13.46	7.26	1.22	7.20	
LLMIC	4.05	2.74	4.31	3.13	4.86	3.89	1.46	3.58	
**Current health expenditure, % of GDP**									< 0.001
HUMIC	6.15	1.97	6.18	2.03	6.96	2.51	0.66	2.43	
LLMIC	5.97	2.67	6.00	2.63	5.24	2.33	0.87	2.18	
**Labour force participation rate, %**^c^									< 0.001
HUMIC	41.41	9.96	41.30	9.87	44.53	12.16	2.47	11.99	
LLMIC	55.57	15.10	54.89	15.40	50.27	14.08	2.44	14.00	
**Prevalence of undernourishment, %**^c^	7.13	6.29	7.13	6.29	5.95	5.79	1.46	5.64	< 0.001
HUMIC	24.06	10.40	23.14	10.46	19.90	11.08	3.16	10.72	
LLMIC									
**Prevalence of HIV/AIDS per 1,000**	8.99	29.89	9.00	29.89	7.06	26.05	0.57	26.22	< 0.001
HUMIC	19.33	24.13	19.26	24.58	16.63	29.86	2.34	30.06	
LLMIC									
**Prevalence of diabetes per 1,000**	72.02	30.13	72.20	30.09	69.66	27.95	2.17	28.06	< 0.001
HUMIC	58.84	16.65	59.70	17.59	66.24	18.79	2.17	18.84	
LLMIC									
**Prevalence of alcohol use disorder per 1,000**	18.21	10.78	18.23	10.78	17.03	9.84	0.40	9.90	0.001
HUMIC	12.06	4.64	12.05	4.61	12.79	5.84	0.37	5.89	
LLMIC									
**Prevalence of daily smoking per 1,000**	172.32	72.60	171.41	72.41	175.50	65.51	8.44	65.40	< 0.001
HUMIC	103.22	54.96	101.56	53.20	124.24	67.45	6.62	67.77	
LLMIC									
**Out-of-pocket health expenditure, % of current health expenditure**	32.09	16.93	32.08	16.95	28.17	16.35	3.39	16.10	< 0.001
HUMIC	44.77	20.03	43.88	20.25	44.02	19.39	4.94	18.93	
LLMIC									
**Case detection rate, % (all forms)**	82.30	8.17	82.30	8.17	83.52	7.36	1.98	7.14	< 0.001
HUMIC	55.38	16.10	56.19	15.37	56.78	16.93	4.93	16.35	
LLMIC									
**Treatment success rate, % (all new cases)**	76.12	13.69	76.26	14.39	76.39	13.83	9.00	10.57	0.001
HUMIC	81.68	8.64	82.25	7.93	82.51	10.67	5.51	9.22	
LLMIC									
**Urbanicity, %**^c^	63.03	16.85	63.10	16.92	67.76	17.99	1.50	18.05	< 0.001
HUMIC	32.28	11.94	32.99	12.68	39.45	14.68	1.61	14.73	
LLMIC	75.43	6.42	75.54	6.64	78.90	8.32	1.70	8.20	

*Abbreviations*: *Obs* Observations, *SD* Standard Deviation, *HIV/AIDS* Human Immunodeficiency Virus/Acquired Immunodeficiency Syndrome, *GDP* Gross Domestic Product, *TB* Tuberculosis, *HUMIC* High- and Upper-Middle Income Country, *LLMIC* Low- and Lower-Middle Income Country

^a^*p*-values describe strength of evidence against a null hypothesis of no difference between country income groups in a t-test

^b^HDI values are multiplied by 100 to ease interpretation of results

^c^Percentages refer to the total population

**Table 3:** Univariable and multivariable associations between social determinants of health and TB incidence rates, stratified by country-income status

	**Univariable analysis**			**Multivariable analysis**		
	**IRR**	**p**	**95%CI**	**IRR**	**p**	**95%CI**
**HDI-within**						
HUMICs	1.0024	0.689	0.9907, 1.0143	1.0009	0.874	0.9894, 1.0127
LLMICs	0.9866	**< .001**	0.9801, 0.9932	0.9870	**< .001**	0.9796, 0.9944
**HDI-between**						
HUMICs	0.8966	**< .001**	0.8782, 0.9153	0.9382	**< .001**	0.9147, 0.9623
LLMICs	0.9469	** < .001**	0.9272, 0.9671	0.9721	**0.010**	0.9515, 0.9933
**Public social protection expenditure-within**						
HUMICs	1.0034	0.646	0.9888, 1.0183	1.0038	0.586	0.9902, 1.0176
LLMICs	1.0022	0.428	0.9967, 1.0078	1.0021	0.573	0.9949, 1.0093
**Public social protection expenditure-between**						
HUMICs	0.9034	**< .001**	0.8827, 0.9246	0.9816	0.236	0.9520, 1.0122
LLMICs	0.9197	0.061	0.8427, 1.0038	0.9379	**0.047**	0.8806, 0.999
**Current health expenditure-within**						
HUMICs	1.0045	0.647	0.9854, 1.024	0.9992	0.919	0.9833, 1.0153
LLMICs	0.9987	0.731	0.9914, 1.006	1.0017	0.668	0.9941, 1.0093
**Current health expenditure-between**						
HUMICs	0.7788	**< .001**	0.7209, 0.8414	0.8931	**< .001**	0.8431, 0.9461
LLMICs	0.9532	0.419	0.8486, 1.0707	0.9781	0.586	0.9032, 1.0592
**Labour force participation rate-within**						
HUMICs	1.0003	0.937	0.9931, 1.0075	1.0015	0.493	0.9971, 1.006
LLMICs	1.0007	0.754	0.9964, 1.005	0.9991	0.671	0.9950, 1.0033
**Labour force participation rate-between**						
HUMICs	0.9692	**0.004**	0.9486, 0.9901	1.0048	0.378	0.9942, 1.0155
LLMICs	1.0153	**0.028**	1.0016, 1.0292	0.9996	0.919	0.9909, 1.0083
**Prevalence of undernourishment-within**						
HUMICs	1.0023	0.369	0.9973, 1.0073	1.0012	0.612	0.9966, 1.0057
LLMICs	1.0020	**0.008**	1.0005, 1.0035	0.9997	0.749	0.9978, 1.0016
**Prevalence of undernourishment-between**						
HUMICs	1.1248	**< .001**	1.1012, 1.1489	0.9809	0.337	0.9429, 1.0203
LLMICs	1.0364	**< .001**	1.0179, 1.0552	1.0059	0.371	0.9930, 1.019
**Prevalence of HIV/AIDS-within**						
HUMICs	1.0076	0.090	0.9988, 1.0165	1.0128	**< .001**	1.0077, 1.018
LLMICs	0.9998	0.932	0.9963, 1.0034	1.0000	0.980	0.9970, 1.003
**Prevalence of HIV/AIDS-between**						
HUMICs	1.0253	**< .001**	1.0171, 1.0335	1.0216	**< .001**	1.0131, 1.0301
LLMICs	1.0130	**< .001**	1.0065, 1.0196	1.0076	**< .001**	1.0043, 1.0109
**Prevalence of diabetes-within**						
HUMICs	1.0107	0.097	0.9981, 1.0234	1.0140	**0.012**	1.0030, 1.0251
LLMICs	1.0032	0.300	0.9971, 1.0094	1.0015	0.590	0.9961, 1.0068
**Prevalence of diabetes-between**						
HUMICs	1.0066	0.059	0.9998, 1.0135	0.9945	**0.039**	0.9893, 0.9997
LLMICs	0.9961	0.478	0.9854, 1.0069	0.9990	0.688	0.9941, 1.0039
**Prevalence of alcohol use disorder-within**						
HUMICs	0.9936	0.841	0.9335, 1.0577	0.9980	0.935	0.9503, 1.0480
LLMICs	0.9955	0.648	0.9762, 1.0151	0.9965	0.769	0.9734, 1.0201
**Prevalence of alcohol use disorder-between**						
HUMICs	1.0247	**0.003**	1.0085, 1.0411	1.0202	**< .001**	1.0083, 1.0323
LLMICs	0.9844	0.503	0.9401, 1.0308	1.0160	0.393	0.9796, 1.0538
**Prevalence of daily smoking-within**						
HUMICs	0.9999	0.906	0.9983, 1.0015	0.9996	0.717	0.9975, 1.0017
LLMICs	1.0005	0.548	0.9990, 1.0019	1.0004	0.540	0.9991, 1.0017
**Prevalence of daily smoking-between**						
HUMICs	0.9973	0.085	0.9941, 1.0004	1.0019	0.066	0.9999, 1.0039
LLMICs	0.9966	**0.009**	0.9941, 0.9992	1.0007	0.604	0.9980, 1.0034
**Out-of-pocket health expenditure-within**						
HUMICs	1.0011	0.664	0.9963, 1.0059	1.0020	0.296	0.9983, 1.0057
LLMICs	1.0006	0.343	0.9994, 1.0018	0.9999	0.933	0.9987, 1.0012
**Out-of-pocket health expenditure-between**						
HUMICs	1.0179	**0.046**	1.0003, 1.0358	1.0060	0.186	0.9971, 1.0149
LLMICs	0.9947	0.294	0.9848, 1.0046	0.9977	0.431	0.9922, 1.0034
**TB case detection rate-within**						
HUMICs	1.0039	0.172	0.9983, 1.0096	1.0029	0.137	0.9991, 1.0067
LLMICs	1.0013	0.062	0.9999, 1.0026	1.0009	0.171	0.9996, 1.0022
**TB case detection rate-between**						
HUMICs	0.9293	**0.008**	0.8806, 0.9807	0.9986	0.863	0.9828, 1.0147
LLMICs	0.9760	**< .001**	0.964, 0.9882	0.9900	**0.038**	0.9806, 0.9994
**Treatment success rate-within**						
HUMICs	0.9997	0.403	0.9989, 1.0005	0.9997	0.433	0.9991, 1.0004
LLMICs	1.0008	**0.016**	1.0001, 1.0014	1.0006	0.061	1, 1.0012
**Treatment success rate-between**						
HUMICs	1.0068	0.483	0.9878, 1.0262	1.0110	0.079	0.9988, 1.0233
LLMICs	0.9622	**< .001**	0.9422, 0.9827	0.9809	**0.013**	0.9661, 0.996
**Urbanicity-within**						
HUMICs	1.0009	0.874	0.9896, 1.0123	1.0042	0.504	0.9919, 1.0167
LLMICs	0.9979	0.763	0.9844, 1.0116	0.9976	0.684	0.9859, 1.0094
**Urbanicity-between**						
HUMICs	0.9700	**< .001**	0.9593, 0.9808	0.9946	0.126	0.9877, 1.0015
LLMICs	0.9814	**0.022**	0.9657, 0.9973	0.9978	0.734	0.9852, 1.0106

N° of observations for HUMICs = 748; N° of observations for LLMICs = 528

Multivariable models controlled for time using year dummies, see Additional file 1: Appendix A.11 and A.12 for coefficients

Bold numbers indicate statistical significance with an acceptable Type I error rate at 5*%*

*Abbreviations*: *SD* Standard Deviation, *CI* Confidence Interval, *IRR* Incidence Rate Ratio, *HIV/AIDS* Human Immunodeficiency Virus/Acquired Immunodeficiency Syndrome, *HDI* Human Development Index, *TB* Tuberculosis, *HUMIC* High- and Upper-Middle Income Country, *LLMIC* Low- and Lower-Middle Income Country

### Multivariable analysis

Results of multivariable within-between regression are presented in Table 3.

#### Within-country

Within LLMICs, increases in HDI over time were associated with lower TB incidence rates. Within HUMICs, increases in the prevalence of diabetes over time were associated with higher TB incidence rates.

#### Between-country

Between LLMICs, higher HDI, public social protection spending, TB case detection rates, and TB treatment success rates were associated with lower TB incidence rates, while higher prevalence of HIV/AIDS was associated with higher TB incidence rates. Between HUMICs, higher HDI, health expenditure spending, and prevalence of diabetes were associated with lower TB incidence rates, while higher prevalence of HIV/AIDS and alcohol use disorder were associated with higher TB incidence rates.

### Sensitivity analysis

We found that results were largely the same as in the main analysis when only including more distal social determinants of health in our multivariable regression. The only significant difference was that within LLMICs, there was no evidence that social protection spending was associated with lower TB incidence rates (Additional file 1: Appendix A.10).

## Discussion

National TB incidence rates decreased in most countries between 2005–2015, declining by a greater proportion in HUMICs compared to LLMICs. Increases observed in HUMICs may have been driven by an increase in prevalence of HIV/AIDS and/or diabetes, whereas increases in LLMICs may have been associated with a slower growth of HDI. In LLMICs, comparing between countries, we find that human development, higher spending on social protection, lower prevalence of HIV/AIDS, and better TB programme performance are significant predictors of lower TB incidence rates. Comparing within-LLMICs, we find a strong link between increases in human development over time and lower TB incidence rates. The pattern appears to be slightly different in HUMICs. Here, comparing between countries, higher human development and spending on healthcare, rather than social protection as in LLMICs, are key predictors of lower TB incidence rates. Our analysis also points to a greater influence of the TB associated comorbidities and health risk behaviours HIV/AIDS, tobacco smoking, and diabetes between-HUMICs relative to between-LLMICs. In addition to lower prevalence of HIV/AIDS, lower prevalence of alcohol use disorder and higher prevalence of diabetes are also significant predictors of lower TB incidence rates. Comparing within-HUMICs, we find a strong link between increases in HIV/AIDS and diabetes prevalence over time and higher TB incidence rates.

To our knowledge, this study provides the most comprehensive insight into the drivers of TB incidence trends at a global level since Dye et al.’s original study in 2009 [17]. More sophisticated statistical methods provide further nuance to our understanding of the drivers of TB incidence and enable us to draw more confident conclusions. The use of a random effects within-between statistical model allows us to evaluate both how variation in determinants between-countries and within-countries over time predicts lower national TB incidence rates.

In relation to existing literature, our findings match evidence that TB disproportionately affects the poorest countries and households, and that human development is a key driver of lower TB incidence [17, 19, 28, 29]. Our findings between-LLMICs are also in line with previous evidence that receipt of social protection, including cash transfers, reduces individuals risk of TB infection and increases their probability of TB treatment success [10, 18, 30]. Evidence that between-HUMICs, health spending is a strong determinant of TB incidence also matches previous evidence [17, 18]. The significance of social protection spending in LLMICs, versus the significance of health spending in HUMICs might indicate a transition in the significance of these determinants as countries develop [31, 32]. However, this would require further research, as distinct from our study, previous evidence supports a positive effect of social protection spending on lower TB incidence rates across Europe [33]. Our findings also add to a large body of literature on the link between both HIV/AIDS and alcohol use and TB incidence [34, 35].

Our contrasting findings for the influence of diabetes prevalence on TB incidence when we compare within- and between-HUMICs, are also consistent with a previous analysis which found that in the same year, diabetes prevalence and TB incidence were inversely related; but that over time, TB incidence was more likely to increase in countries where diabetes prevalence increased [36]. Diabetes is more common in richer countries that have better developed health systems and lower levels of disease risk factors that are positively associated with TB incidence, such as environmental pollutants [37]. Our findings that as the prevalence of diabetes increases declines in TB incidence slow could be explained by diabetes and TB interacting biologically [38], and/or countries national control of TB incidence being temporarily affected by an increasing need to simultaneously respond to growing numbers of diabetes patients. Diabetes management poses a significant financial burden to health systems and could affect funds available for other health priorities [39, 40]. The process of social development involves significant changes in demography, the distribution of risk factors, and organisation and quality of health services [41]*,* and further analysis would be needed to unpack the precise mechanisms underlying the observed within-country association between diabetes prevalence and TB incidence.

For TB programme performance, we find that in LLMICs, between-countries, those with higher TB programme performance have lower TB incidence rates. However, similar to Dye et al., we still find no evidence in either LLMICs or HUMICs that increases in TB control performance over time are associated with lower TB incidence [17]. Further research is needed to understand how investments in this area can lead to much needed impacts on TB transmission [17].

This study had a number of strengths. Data were selected from the most comprehensive online sources and represent the best available data today. The use of within-between random effects specifications allowed us to evaluate both, more causally robust within-country relationships, and policy relevant between-country relationships. This methodological approach builds on Dye et al.’s evaluation of incidence trends as 10-year averages. The study also has limitations. First, like Dye et al. it relies on estimated values of TB incidence, TB treatment success, and TB case detection from national TB surveillance systems [17]. All associations were also investigated at the population level, and as such should not be interpreted as causal or as applying to the individual level [42]. Excluding countries with missing data at baseline affects the generalizability of our findings to these countries. Nevertheless, inclusion of 24/30 high TB burden countries gives confidence that our results are likely to apply in settings where action to reduce TB incidence is most needed [1]. The range of predictors in our model, also resulted in very small coefficients for some variables like prevalence of HIV/AIDS and made it difficult to compare effect sizes. Finally, we also had to exclude some predictors that could have provided further insight into preventing TB due to high levels of missing data including proportion of urban population living in slums, coverage of social protection and labour programmes, and total TB expenditure.

In the shadow of the global COVID-19 crisis which threatens to reverse decade long gains in development [43], intensified innovation and cost-saving solutions will be required to achieve End TB Strategy goals by 2030. Largely consistent with Dye et al.’s findings from 2009, this study provides updated evidence that indicators of human and social development may be stronger determinants of TB incidence decline than indicators of TB programme performance, especially in LLMICs [17]. Our study also reinforces the positive impact that actions to prevent rising rates of HIV/AIDS and diabetes could have on reducing TB incidence rates in HUMICs [2]. As promoted by the WHO, this could include intensified collaborative activities on diabetes and tuberculosis prevention, including integration of TB control strategies in broader health interventions that target non-communicable diseases [44]. Action on diabetes prevention in countries of lower socioeconomic status might become more urgent considering the projected increases in diabetes prevalence in low- and middle-income countries by 0.7–2.3% between 2019 and 2045, where most of the global TB burden is concentred today [21, 45, 46]. To maximise reductions in global TB incidence, investments should be targeted to countries in most need of support. Findings from our between-country analysis point to a number of indicators including low human development and social protection spending, high HIV prevalence and alcohol use, and poor TB programme performance that could be used to target strategic investments to reduce TB incidence globally [47].

In the future, research is needed to understand how national TB programmes can support the implementation of cost-effective approaches to improve human development in LLMICs, and prevent HIV/AIDS, and diabetes in HUMICs. It would be important for such efforts to consider the spill over effects of action on the social determinants of TB for other disease prevention programmes, especially those focused on HIV/AIDS and diabetes.

## Conclusion

In LLMICs, TB incidence rates remain highest in countries with low human development, social protection spending, and TB programme performance, and high rates of HIV/AIDS. Strengthening human development is likely to accelerate declines in TB incidence. In HUMICs, TB incidence rates remain highest in countries with low human development, health spending, and diabetes prevalence, and high rates of HIV/AIDS and alcohol use. Here, slowing rising rates of HIV/AIDS and diabetes is likely to accelerate declines in TB incidence. Further research should focus on which investments are most likely to translate into change in these areas.

## Supplementary Information

**Additional file 1.** 

## Data Availability

The datasets generated and/or analysed during the current study are available in the Open Science Framework repository, https://osf.io/x6uag/

## Abbreviations

TB: Tuberculosis
WHO: World Health Organization
HIV: Human Immunodeficiency Virus
AIDS: Acquired Immune Deficiency Syndrome
HDI: Human Development Index
HUCI: High- and upper-middle-income country
LLMIC: Low- and lower-middle-income country
SDG: Sustainable Development Goals
CSDH: Commission on Social Determinants of Health
U5M: Under-5 mortality
MDG: Millennium Development Goals
GDB: Global Burden of Disease Study
HDR: Human Development Report
STROBE: Strengthening the reporting of observational studies in epidemiology
ILO: International Labour Organization
IRR: Incidence rate ratio
SD: Standard deviation
IQR: Interquartile range
CI: Confidence interval
ART: Antiretroviral therapy
