# Supplementary material for: The social determinants of national tuberculosis incidence rates in 116 countries: a longitudinal ecological study between 2005–2015
Source: BMC Public Health. 2023 Feb 15;23:337. doi: 10.1186/s12889-023-15213-w (PMC9930041; doi:10.1186/s12889-023-15213-w)
Supplement: Supplementary file 1 — Additional file 1. [file 12889_2023_15213_MOESM1_ESM.docx]

**Appendix**

**A.1 STROBE Statement—checklist of items that should be included in reports of observational studies.**

|  | Item No. | Recommendation | Page  No. | Relevant text from manuscript |
| --- | --- | --- | --- | --- |
| **Title and abstract** | 1 | (*a*) Indicate the study’s design with a commonly used term in the title or the abstract | 1 | “The Social Determinants of National Tuberculosis Incidence Rates in 116 Countries: A Longitudinal Ecological Study between 2005-2015” |
|  |  | (*b*) Provide in the abstract an informative and balanced summary of what was done and what was found | 2 | Abstract sections Methods and Conclusion |
| Introduction | | | |  |
| Background/rationale | 2 | Explain the scientific background and rationale for the investigation being reported | 4 | Line 9-33 |
| Objectives | 3 | State specific objectives, including any prespecified hypotheses | 5 | Line 34-45 |
| Methods | | | |  |
| Study design | 4 | Present key elements of study design early in the paper | 6 | Line 46-51 |
| Setting | 5 | Describe the setting, locations, and relevant dates, including periods of recruitment, exposure, follow-up, and data collection | 6 | Line 53-56 |
| Participants | 6 | (*a*) *Cohort study*—Give the eligibility criteria, and the sources and methods of selection of participants. Describe methods of follow-up  *Case-control study*—Give the eligibility criteria, and the sources and methods of case ascertainment and control selection. Give the rationale for the choice of cases and controls  *Cross-sectional study*—Give the eligibility criteria, and the sources and methods of selection of participants |  | Not applicable |
|  |  | (*b*) *Cohort study*—For matched studies, give matching criteria and number of exposed and unexposed  *Case-control study*—For matched studies, give matching criteria and the number of controls per case |  | Not applicable |
| Variables | 7 | Clearly define all outcomes, exposures, predictors, potential confounders, and effect modifiers. Give diagnostic criteria, if applicable | 7, 17, A.5-6 | Line 71-85, Table 1, A.3 |
| Data sources/ measurement | 8* | For each variable of interest, give sources of data and details of methods of assessment (measurement). Describe comparability of assessment methods if there is more than one group | 7, 17 | Line 72-85, Table 1 |
| Bias | 9 | Describe any efforts to address potential sources of bias | 6, 7, 8 | Line 54-69, 71, 78-84, 86-90 |
| Study size | 10 | Explain how the study size was arrived at | 6, 7, 10 | Line 53-69, Line 117-122, Figure 2 |
| Quantitative variables | 11 | Explain how quantitative variables were handled in the analyses. If applicable, describe which groupings were chosen and why | 6, 7 | Line 71-85 |
| Statistical methods | 12 | (*a*) Describe all statistical methods, including those used to control for confounding | 7, 8 | Line 80-85, Line 91-109 |
|  |  | (*b*) Describe any methods used to examine subgroups and interactions |  | Not applicable |
|  |  | (*c*) Explain how missing data were addressed | 7 | Line 86-90 |
|  |  | (*d*) *Cohort study*—If applicable, explain how loss to follow-up was addressed  *Case-control study*—If applicable, explain how matching of cases and controls was addressed  *Cross-sectional study*—If applicable, describe analytical methods taking account of sampling strategy |  | Not applicable |
|  |  | (*e*) Describe any sensitivity analyses | 8 | Line 111-116 |
| Results | | | | |
| Participants | 13* | (a) Report numbers of individuals at each stage of study—eg numbers potentially eligible, examined for eligibility, confirmed eligible, included in the study, completing follow-up, and analysed | 9,10 | Line 117-122, Figure 2 |
|  |  | (b) Give reasons for non-participation at each stage | 9 | Line 117-119 |
|  |  | (c) Consider use of a flow diagram | 10 | Figure 2 |
| Descriptive data | 14* | (a) Give characteristics of study participants (eg demographic, clinical, social) and information on exposures and potential confounders | 10, 18-19 | Line 125-137, Table 2 |
|  |  | (b) Indicate number of participants with missing data for each variable of interest |  | Not applicable |
|  |  | (c) *Cohort study*—Summarise follow-up time (eg, average and total amount) |  | Not applicable |
| Outcome data | 15* | *Cohort study*—Report numbers of outcome events or summary measures over time |  | Not applicable |
|  |  | *Case-control study—*Report numbers in each exposure category, or summary measures of exposure |  | Not applicable |
|  |  | *Cross-sectional study—*Report numbers of outcome events or summary measures |  | Not applicable |
| Main results | 16 | (*a*) Give unadjusted estimates and, if applicable, confounder-adjusted estimates and their precision (eg, 95% confidence interval). Make clear which confounders were adjusted for and why they were included | 7 | 138-146, Table 3 |
|  |  | (*b*) Report category boundaries when continuous variables were categorized |  | Not applicable |
|  |  | (*c*) If relevant, consider translating estimates of relative risk into absolute risk for a meaningful time period |  | Not applicable |
| Other analyses | 17 | Report other analyses done—eg analyses of subgroups and interactions, and sensitivity analyses | 11 | Line 147-150 |
| Discussion | | | | |
| Key results | 18 | Summarise key results with reference to study objectives | 11, 12 | Line 152-168 |
| Limitations | 19 | Discuss limitations of the study, taking into account sources of potential bias or imprecision. Discuss both direction and magnitude of any potential bias | 14 | Line 209-225 |
| Interpretation | 20 | Give a cautious overall interpretation of results considering objectives, limitations, multiplicity of analyses, results from similar studies, and other relevant evidence | 11-14 | Line 169-208 |
| Generalisability | 21 | Discuss the generalisability (external validity) of the study results | 14 | Line 217-220 |
| Other information |  | | | |
| Funding | 22 | Give the source of funding and the role of the funders for the present study and, if applicable, for the original study on which the present article is based | 25 | Declarations: Funding |

**A.2** **Scatterplot summarising trends in TB incidence between 2005-2015 by World Bank country-income classifications.**

Abbreviations: TB=Tuberculosis.

**A.3 List of included countries.**

| **Country** | **2005 WB Income Classification** |
| --- | --- |
| Afghanistan | L |
| Algeria | LM |
| Angola | LM |
| Armenia | LM |
| Australia | H |
| Austria | H |
| Azerbaijan | LM |
| Bangladesh | L |
| Belarus | LM |
| Belgium | H |
| Belize | UM |
| Benin | L |
| Bolivia | LM |
| Botswana | UM |
| Brazil | LM |
| Bulgaria | LM |
| Burkina Faso | L |
| Cambodia | L |
| Cameroon | LM |
| Canada | H |
| Central African Republic | L |
| Chile | UM |
| China | LM |
| Colombia | LM |
| Congo | LM |
| Costa Rica | UM |
| Cote d'Ivoire | L |
| Croatia | UM |
| Cuba | LM |
| Cyprus | H |
| Czech Republic | UM |
| Denmark | H |
| Dominican Republic | LM |
| Ecuador | LM |
| Egypt | LM |
| El Salvador | LM |
| Estonia | UM |
| Fiji | LM |
| Georgia | LM |
| Germany | H |
| Ghana | L |
| Guinea | L |
| Guyana | LM |
| Honduras | LM |
| Hungary | UM |
| Iceland | H |
| India | L |
| Indonesia | LM |
| Iran | LM |
| Ireland | H |
| Israel | H |
| Jamaica | LM |
| Japan | H |
| Jordan | LM |
| Kazakhstan | LM |
| Kenya | L |
| Kuwait | H |
| Kyrgyzstan | L |
| Laos | L |
| Latvia | UM |
| Lesotho | LM |
| Lithuania | UM |
| Madagascar | L |
| Malaysia | UM |
| Maldives | LM |
| Malta | H |
| Mauritania | L |
| Mauritius | UM |
| Mexico | UM |
| Mongolia | L |
| Morocco | LM |
| Mozambique | L |
| Myanmar | L |
| Namibia | LM |
| Nepal | L |
| Netherlands | H |
| New Zealand | H |
| Nicaragua | LM |
| Niger | L |
| Nigeria | L |
| Oman | UM |
| Pakistan | L |
| Panama | UM |
| Paraguay | LM |
| Peru | LM |
| Philippines | LM |
| Poland | UM |
| Portugal | H |
| Romania | UM |
| Russian Federation | UM |
| Rwanda | L |
| Samoa | LM |
| Senegal | L |
| Sierra Leone | L |
| Slovakia | UM |
| Slovenia | H |
| Solomon Islands | L |
| South Africa | UM |
| Sri Lanka | LM |
| Sweden | H |
| Tanzania | L |
| Thailand | LM |
| The Gambia | L |
| Timor-Leste | L |
| Togo | L |
| Trinidad and Tobago | UM |
| Tunisia | LM |
| Uganda | L |
| United Kingdom | H |
| United States | H |
| Uruguay | UM |
| Uzbekistan | L |
| Venezuela | UM |
| Vietnam | L |
| Yemen | L |
| Zambia | L |

Abbreviations: WB=World Bank; L= Low Income Country; LM=Lower-Middle Income Country; UM=Upper-Middle Income Country; H=High Income Country.

**A.4 List of excluded countries.**

| American Samoa | Northern Mariana Islands |
| --- | --- |
| Andorra | Norway |
| Antigua and Barbuda | Palestine |
| Argentina | Papua New Guinea |
| Bahrain | Puerto Rico |
| Barbados | Qatar |
| Bermuda | Saint Lucia |
| Bhutan | Saint Vincent and the Grenadines |
| Bosnia and Herzegovina | Sao Tome and Principe |
| Brunei | Saudi Arabia |
| Burundi | Serbia |
| Cape Verde | Seychelles |
| Chad | Singapore |
| Comoros | Somalia |
| Democratic Republic of the Congo | South Korea |
| Djibouti | South Sudan |
| Dominica | Spain |
| Ethiopia | Sudan |
| Equatorial Guinea | Suriname |
| Eritrea | Swaziland |
| Federated States of Micronesia | Switzerland |
| Finland | Syria |
| France | Taiwan (Province of China) |
| Gabon | Tajikistan |
| Greece | The Bahamas |
| Greenland | Tonga |
| Grenada | Turkey |
| Guam | Turkmenistan |
| Guatemala | Ukraine |
| Guinea-Bissau | United Arab Emirates |
| Haiti | Vanuatu |
| Iraq | Virgin Islands, U.S. |
| Italy | Zimbabwe |
| Kiribati |  |
| Lebanon |  |
| Liberia |  |
| Libya |  |
| Luxembourg |  |
| Macedonia |  |
| Malawi |  |
| Mali |  |
| Marshall Islands |  |
| Moldova |  |
| Montenegro |  |
| North Korea |  |

**A.5 Correlation matrix for within-country variation across seventeen indicators considered for inclusion in the analysis, all countries.**

|  | Current health expenditure | Public social protection expenditure | Labour force participation rate | TB case detection rate | TB treatment success rates | Population with access to clean fuels and technologies for cooking, % | Out-of-pocket health expenditure | Population with access to basic sanitation services, % | Population with access to basic drinking water services, % | Under-5 mortality rate | Prevalence of undernourishment | Urbanicity | Prevalence of HIV/AIDS per 1,000 | Prevalence of alcohol use disorder per 1,000 | Prevalence of diabetes per 1,000 | Prevalence of daily smoking per 1,000 | Human Development Index |
| --- | --- | --- | --- | --- | --- | --- | --- | --- | --- | --- | --- | --- | --- | --- | --- | --- | --- |
| Current health expenditure | 1.00 |  |  |  |  |  |  |  |  |  |  |  |  |  |  |  |  |
| Public social protection expenditure | 0.31 | 1.00 |  |  |  |  |  |  |  |  |  |  |  |  |  |  |  |
| Labour force participation rate | -0.13 | -0.34 | 1.00 |  |  |  |  |  |  |  |  |  |  |  |  |  |  |
| TB case detection rate | 0.07 | 0.09 | -0.22 | 1.00 |  |  |  |  |  |  |  |  |  |  |  |  |  |
| TB treatment success rates | 0.03 | 0.03 | 0.01 | 0.01 | 1.00 |  |  |  |  |  |  |  |  |  |  |  |  |
| Population with access to clean fuels and technologies for cooking | 0.17 | 0.23 | -0.15 | 0.23 | 0.06 | 1.00 |  |  |  |  |  |  |  |  |  |  |  |
| Out-of-pocket health expenditure | -0.16 | -0.12 | 0.22 | -0.06 | -0.02 | -0.21 | 1.00 |  |  |  |  |  |  |  |  |  |  |
| Population with access to basic sanitation services | 0.17 | 0.32 | -0.34 | 0.31 | 0.09 | 0.62 | -0.19 | 1.00 |  |  |  |  |  |  |  |  |  |
| Population with access to basic drinking water services | 0.13 | 0.31 | -0.30 | 0.24 | 0.13 | 0.53 | -0.22 | 0.66 | 1.00 |  |  |  |  |  |  |  |  |
| Under-5 mortality rate | -0.02 | -0.22 | 0.41 | -0.38 | -0.15 | -0.44 | 0.25 | -0.61 | -0.65 | 1.00 |  |  |  |  |  |  |  |
| Prevalence of undernourishment | -0.18 | -0.16 | 0.27 | -0.16 | 0.00 | -0.42 | 0.18 | -0.43 | -0.48 | 0.48 | 1.00 |  |  |  |  |  |  |
| Urbanicity | 0.13 | 0.30 | -0.35 | 0.27 | 0.16 | 0.53 | -0.29 | 0.60 | 0.59 | -0.60 | -0.39 | 1.00 |  |  |  |  |  |
| Prevalence of HIV/AIDS per 1,000 | 0.20 | 0.11 | 0.00 | -0.12 | -0.06 | 0.05 | 0.04 | 0.02 | -0.04 | 0.17 | -0.05 | -0.11 | 1.00 |  |  |  |  |
| Prevalence of alcohol use disorder per 1,000 | 0.13 | 0.15 | -0.05 | 0.02 | 0.04 | 0.12 | -0.01 | 0.09 | 0.08 | -0.06 | -0.09 | 0.10 | 0.02 | 1.00 |  |  |  |
| Prevalence of diabetes per 1,000 | 0.20 | 0.30 | -0.29 | 0.22 | 0.03 | 0.50 | -0.21 | 0.47 | 0.47 | -0.46 | -0.34 | 0.53 | -0.02 | 0.17 | 1.00 |  |  |
| Prevalence of daily smoking per 1,000 | -0.27 | -0.28 | 0.26 | -0.16 | -0.02 | -0.28 | 0.01 | -0.40 | -0.29 | 0.27 | 0.22 | -0.41 | -0.05 | -0.11 | -0.33 | 1.00 |  |
| Human Development Index | 0.20 | 0.37 | -0.41 | 0.30 | 0.11 | 0.61 | -0.30 | 0.71 | 0.68 | -0.78 | -0.54 | 0.73 | -0.10 | 0.14 | 0.63 | -0.46 | 1.00 |

Abbreviations: TB=Tuberculosis; HIV=Human Immunodeficiency Virus/Acquired Immunodeficiency Syndrome.

**A.6 Correlation matrix for between-country variation across seventeen indicators considered for inclusion in the analysis, all countries.**

|  | Current health expenditure | Public social protection expenditure | Labour force participation rate | TB case detection rate | TB treatment success rates | Population with access to clean fuels and technologies for cooking, % | Out-of-pocket health expenditure | Population with access to basic sanitation services, % | Population with access to basic drinking water services, % | Under-5 mortality rate | Prevalence of undernourishment | Urbanicity | Prevalence of HIV/AIDS per 1,000 | Prevalence of alcohol use disorder per 1,000 | Prevalence of diabetes per 1,000 | Prevalence of daily smoking per 1,000 | Human Development Index |
| --- | --- | --- | --- | --- | --- | --- | --- | --- | --- | --- | --- | --- | --- | --- | --- | --- | --- |
| Current health expenditure | 1.00 |  |  |  |  |  |  |  |  |  |  |  |  |  |  |  |  |
| Public social protection expenditure | 0.61 | 1.00 |  |  |  |  |  |  |  |  |  |  |  |  |  |  |  |
| Labour force participation rate | 0.14 | -0.06 | 1.00 |  |  |  |  |  |  |  |  |  |  |  |  |  |  |
| TB case detection rate | 0.41 | 0.60 | -0.13 | 1.00 |  |  |  |  |  |  |  |  |  |  |  |  |  |
| TB treatment success rates | -0.09 | -0.27 | 0.08 | -0.20 | 1.00 |  |  |  |  |  |  |  |  |  |  |  |  |
| Population with access to clean fuels and technologies for cooking | 0.37 | 0.71 | -0.28 | 0.77 | -0.23 | 1.00 |  |  |  |  |  |  |  |  |  |  |  |
| Out-of-pocket health expenditure | -0.33 | -0.49 | -0.11 | -0.37 | 0.18 | -0.31 | 1.00 |  |  |  |  |  |  |  |  |  |  |
| Population with access to basic sanitation services | 0.31 | 0.63 | -0.25 | 0.77 | -0.13 | 0.90 | -0.32 | 1.00 |  |  |  |  |  |  |  |  |  |
| Population with access to basic drinking water services | 0.26 | 0.58 | -0.29 | 0.70 | -0.08 | 0.84 | -0.30 | 0.88 | 1.00 |  |  |  |  |  |  |  |  |
| Under-5 mortality rate | -0.30 | -0.60 | 0.13 | -0.76 | 0.03 | -0.81 | 0.38 | -0.88 | -0.87 | 1.00 |  |  |  |  |  |  |  |
| Prevalence of undernourishment | -0.28 | -0.58 | 0.26 | -0.62 | 0.07 | -0.73 | 0.16 | -0.76 | -0.79 | 0.68 | 1.00 |  |  |  |  |  |  |
| Urbanicity | 0.35 | 0.65 | -0.16 | 0.58 | -0.19 | 0.78 | -0.37 | 0.66 | 0.65 | -0.60 | -0.56 | 1.00 |  |  |  |  |  |
| Prevalence of HIV/AIDS per 1,000 | 0.01 | -0.15 | 0.05 | -0.31 | -0.09 | -0.32 | -0.20 | -0.40 | -0.37 | 0.40 | 0.43 | -0.24 | 1.00 |  |  |  |  |
| Prevalence of alcohol use disorder per 1,000 | 0.08 | 0.31 | -0.05 | 0.30 | -0.13 | 0.26 | -0.16 | 0.24 | 0.23 | -0.27 | -0.17 | 0.16 | -0.02 | 1.00 |  |  |  |
| Prevalence of diabetes per 1,000 | -0.17 | -0.15 | -0.14 | 0.05 | -0.07 | -0.03 | -0.02 | 0.09 | 0.09 | -0.04 | -0.01 | -0.14 | 0.10 | -0.17 | 1.00 |  |  |
| Prevalence of daily smoking per 1,000 | 0.17 | 0.48 | -0.16 | 0.35 | -0.03 | 0.41 | -0.20 | 0.48 | 0.50 | -0.53 | -0.41 | 0.23 | -0.25 | 0.31 | 0.01 | 1.00 |  |
| Human Development Index | 0.40 | 0.77 | -0.15 | 0.74 | -0.18 | 0.89 | -0.44 | 0.90 | 0.87 | -0.90 | -0.73 | 0.76 | -0.35 | 0.27 | -0.03 | 0.53 | 1.00 |

Abbreviations: TB=Tuberculosis; HIV=Human Immunodeficiency Virus/Acquired Immunodeficiency Syndrome.

**A.7 Definitions of independent study variables included in the study.**

Human Development Index: The Human Development Index (HDI) is a summary measure of average achievement in key dimensions of human development: a long and healthy life, being knowledgeable and have a decent standard of living. The HDI is the geometric mean of normalized indices for each of the three dimensions.

**Public social protection expenditure, % of GDP:** Total public social protection expenditure, including health, percentage of GDP. Data on social protection expenditure are collected according to different standards around the world.

Current health expenditure per capita, PPP (current international $): Current expenditures on health per capita expressed in international dollars at purchasing power parity (PPP)

Labour force participation rate, % of total population aged 15-64: Labour force participation rate is the proportion of the population ages 15-64 that is economically active: all people who supply labour for the production of goods and services during a specified period.

**Prevalence of undernourishment, %:** Population below minimum level of dietary energy consumption (also referred to as prevalence of undernourishment) shows the percentage of the population whose food intake is insufficient to meet dietary energy requirements continuously.

**Prevalence of HIV/AIDS, per 1,000 (age-standardized, both sexes):** Case definition according to ICD-10 codes: B20-B23.8, B24-B24.0, B97.81, C46-C46.52, C46.7-C46.9, O98.7-O98.73, Z11.4, Z20.6, Z21, Z22.6, Z83.0.

**Prevalence of diabetes, per 1,000 (age-standardized, both sexes):** Case definition according to ICD-10 codes: E08-E08.11, E08.3-E08.9, E12-E12.1, E12.3-E13.11, E13.3-E14.1, E14.3-E14.9, R73-R73.9.

**Prevalence of alcohol use disorder, per 1,000 (age-standardized, both sexes):** Case definition according to ICD-10 codes: F10-F10.99, G31.2, R78.0, X45-X45.9, X65-X65.9, Y15-Y15.9, Z81.1.

**Prevalence of daily smoking, per 1,000 (age-standardized, both sexes):** Individual using any type of smoked tobacco product on a daily basis.

**Out-of-pocket expenditure, % of current health expenditure:** Share of out-of-pocket payments of total current health expenditures. Out-of-pocket payments are spending on health directly out-of-pocket by households.

**Treatment success rate for all new cases (%):** Treatment success rate for all new cases (including relapse cases).

**Case detection rate, % (all forms):** Case detection rate (all forms) [also known as TB treatment coverage], percent.

Population living in urban areas, %: Urban population refers to people living in urban areas as defined by national statistical offices. The data are collected and smoothed by United Nations Population Division.

**A.8 Characteristics of observed vs. interpolated study variables.**

| **Observed variable** | **N°** | **Mean** | **SD** | **Min** | **Max** |
| --- | --- | --- | --- | --- | --- |
| Human Development Index | 1,276 | 68.22 | 15.54 | 28.30 | 93.30 |
| Public social protection expenditure | 520 | 11.23 | 7.91 | 0.10 | 29.32 |
| Current health expenditure | 1,276 | 6.25 | 2.58 | 1.03 | 20.41 |
| Labour force participation rate | 1,276 | 46.91 | 13.29 | 23.00 | 79.95 |
| Out-of-pocket health expenditure | 1,276 | 34.73 | 19.31 | 3.43 | 87.10 |
| Population with access to clean fuels and technologies for cooking | 1,276 | 62.85 | 37.38 | 0.27 | 100.00 |
| Population with access to basic sanitation services | 1,276 | 71.30 | 29.68 | 6.15 | 100.00 |
| Population with access to basic drinking water sources | 1,276 | 84.85 | 17.71 | 29.41 | 100.00 |
| Under-5 mortality rate | 1,276 | 37.71 | 38.69 | 2.20 | 203.60 |
| Prevalence of undernourishment | 1,276 | 11.72 | 10.84 | 2.50 | 57.80 |
| Urbanicity | 1,276 | 56.05 | 21.75 | 15.15 | 100.00 |
| TB treatment success rate | 1,257 | 78.98 | 12.40 | 0.00 | 100.00 |
| TB case detection rate | 1,272 | 72.46 | 17.97 | 15.00 | 110.00 |
| Prevalence of HIV per 1,000 | 1,276 | 11.02 | 28.08 | 0.02 | 173.69 |
| Prevalence of alcohol use disorder per 1,000 | 1,276 | 15.28 | 8.67 | 4.68 | 51.23 |
| Prevalence of diabetes per 1,000 | 1,276 | 68.25 | 24.63 | 31.42 | 218.72 |
| Prevalence of daily smoking per 1,000 | 1,276 | 154.29 | 70.94 | 33.08 | 349.83 |
| **Interpolated variable** | **N°** | **Mean** | **SD** | **Min** | **Max** |
| Human Development Index | 1,276 | 68.22 | 15.54 | 28.30 | 93.30 |
| Public social protection expenditure | 1,256 | 9.98 | 7.44 | 0.10 | 29.32 |
| Current health expenditure | 1,276 | 6.25 | 2.58 | 1.03 | 20.41 |
| Labour force participation rate | 1,276 | 46.91 | 13.29 | 23.00 | 79.95 |
| Out-of-pocket health expenditure | 1,276 | 34.73 | 19.31 | 3.43 | 87.10 |
| Population with access to clean fuels and technologies for cooking | 1,276 | 62.85 | 37.38 | 0.27 | 100.00 |
| Population with access to basic sanitation services | 1,276 | 71.30 | 29.68 | 6.15 | 100.00 |
| Population with access to basic drinking water sources | 1,276 | 84.85 | 17.71 | 29.41 | 100.00 |
| Under-5 mortality rate | 1,276 | 37.71 | 38.69 | 2.20 | 203.60 |
| Prevalence of undernourishment | 1,276 | 11.72 | 10.84 | 2.50 | 57.80 |
| Urbanicity | 1,276 | 56.05 | 21.75 | 15.15 | 100.00 |
| TB treatment success rate | 1,276 | 78.93 | 12.96 | 0.00 | 180.00 |
| TB case detection rate | 1,276 | 72.46 | 17.99 | 15.00 | 110.00 |
| Prevalence of HIV per 1,000 | 1,276 | 11.02 | 28.08 | 0.02 | 173.69 |
| Prevalence of alcohol use disorder per 1,000 | 1,276 | 15.28 | 8.67 | 4.68 | 51.23 |
| Prevalence of diabetes per 1,000 | 1,276 | 68.25 | 24.63 | 31.42 | 218.72 |
| Prevalence of daily smoking per 1,000 | 1,276 | 154.29 | 70.94 | 33.08 | 349.83 |

Abbreviations: SD=Standard Deviation; TB=Tuberculosis; HIV=Human Immunodeficiency Virus/Acquired Immunodeficiency Syndrome.

Abbreviations: TB=Tuberculosis; HUMIC= High- and Upper-Middle Income Country; LLMIC=Low- and Lower-Middle Income Country.

**A.9 Distribution of TB incidence per 100,000 in LLMIC and HUMIC.**

**A.10 Sensitivity analysis of multivariable associations between structural social determinants of health and TB incidence rates.**

|  |  | | |
| --- | --- | --- | --- |
|  | **IRR** | **p** | **95%CI** |
| **HDI-within** |  |  |  |
| HUMICs | 1.0034 | 0.670 | 0.9879, 1.0191 |
| LLMICs | 0.9859 | **<.001** | 0.9791, 0.9928 |
| **HDI-between** |  |  |  |
| HUMICs | 0.8952 | **<.001** | 0.8586, 0.9334 |
| LLMICs | 0.9484 | **<.001** | 0.9284, 0.9688 |
| **Public social protection expenditure-within** |  |  |  |
| HUMICs | 1.0039 | 0.639 | 0.9876, 1.0205 |
| LLMICs | 1.0014 | 0.655 | 0.9953, 1.0075 |
| **Public social protection expenditure-between** |  |  |  |
| HUMICs | 1.0245 | 0.156 | 0.9908, 1.0593 |
| LLMICs | 0.9854 | 0.682 | 0.9186, 1.0571 |
| **Current health expenditure-within** |  |  |  |
| HUMICs | 1.0050 | 0.578 | 0.9875, 1.0228 |
| LLMICs | 1.0035 | 0.388 | 0.9956, 1.0115 |
| **Current health expenditure-between** |  |  |  |
| HUMICs | 0.9001 | **0.027** | 0.8199, 0.9881 |
| LLMICs | 0.9219 | 0.085 | 0.8404, 1.0113 |
| **Labour force participation rate-within** |  |  |  |
| HUMICs | 1.0007 | 0.864 | 0.9931, 1.0083 |
| LLMICs | 0.9990 | 0.631 | 0.995, 1.003 |
| **Labour force participation rate-between** |  |  |  |
| HUMICs | 1.0001 | 0.992 | 0.9865, 1.0138 |
| LLMICs | 1.0056 | 0.304 | 0.995, 1.0162 |

Abbreviations: IRR=Incidence Rate Ratio; CI=Confidence Interval; HDI=Human Development Index; HUMIC= High- and Upper-Middle Income Country; LLMIC=Low- and Lower-Middle Income Country.

**A.11 Year dummies for LLMICs in multivariable within-between regression analysis.**

| year | IRR | p | 95% CI |
| --- | --- | --- | --- |
| 2006 | 0.9974 | 0.662 | 0.9858, 1.0091 |
| 2007 | 0.9897 | 0.359 | 0.9681, 1.0118 |
| 2008 | 0.9812 | 0.259 | 0.9495, 1.0140 |
| 2009 | 0.9734 | 0.210 | 0.9332, 1.0153 |
| 2010 | 0.9691 | 0.226 | 0.9210, 1.0197 |
| 2011 | 0.9684 | 0.283 | 0.9132, 1.0269 |
| 2012 | 0.9670 | 0.325 | 0.9045, 1.0338 |
| 2013 | 0.9652 | 0.355 | 0.8953, 1.0405 |
| 2014 | 0.9638 | 0.381 | 0.8874, 1.0467 |
| 2015 | 0.9648 | 0.437 | 0.8813, 1.0561 |

Abbreviations: IRR=Incidence Rate Ratio; CI=Confidence Interval; LLMIC=Low- and Lower-Middle Income Country.

**A.12 Year dummies for HUMICs in multivariable within-between regression analysis.**

| year | IRR | p | 95%CI |
| --- | --- | --- | --- |
| 2006 | 0.9798 | 0.003 | 0.9665, 0.9932 |
| 2007 | 0.9561 | <.001 | 0.9338, 0.9789 |
| 2008 | 0.9237 | <.001 | 0.8905, 0.9581 |
| 2009 | 0.8986 | <.001 | 0.8594, 0.9395 |
| 2010 | 0.8793 | <.001 | 0.8333, 0.9278 |
| 2011 | 0.8558 | <.001 | 0.8024, 0.9127 |
| 2012 | 0.8369 | <.001 | 0.7821, 0.8955 |
| 2013 | 0.8081 | <.001 | 0.7449, 0.8766 |
| 2014 | 0.7843 | <.001 | 0.7138, 0.8617 |
| 2015 | 0.7560 | <.001 | 0.6771, 0.8440 |

Abbreviations: IRR=Incidence Rate Ratio; CI=Confidence Interval; HUMIC= High- and Upper-Middle Income Country.
